# Association between polypharmacy at the emergency department and long-term mortality in critically ill older patients receiving mechanical ventilation: a single-center retrospective cohort study

**DOI:** 10.1186/s12873-025-01463-x

**Published:** 2026-01-10

**Authors:** Yoshihiro Nakamura, Takeshi Umegaki, Kota Nishimoto, Takashi Muroya, Takahiko Kamibayashi, Yasuyuki Kuwagata

**Affiliations:** 1 Department of Emergency and Critical Care Medicine, Kansai Medical University, 2-5-1 Shin-machi, Hirakata, Osaka 573-1010 Japan; 2 Department of Anesthesiology, Kansai Medical University, 2-5-1 Shin-machi, Hirakata, Osaka 573-1010 Japan

**Keywords:** Polypharmacy, Critical illness, Older adults, Mortality, Emergency medical services

## Abstract

**Background:**

Polypharmacy is increasingly prevalent among older adults, and is associated with adverse health outcomes. However, its prognostic impact in emergency care settings remains unclear, particularly in critically ill older patients requiring mechanical ventilation. Therefore, this study aimed to evaluate the association between polypharmacy at the emergency department and long-term mortality in critically ill older patients who required mechanical ventilation.

**Methods:**

We conducted a retrospective cohort study of emergency department patients aged ≥ 65 years who received mechanical ventilation at a Japanese university hospital between April 2015 and December 2024. Patients were categorized into a polypharmacy group (≥ 5 regular medications at admission) or a non-polypharmacy group (fewer medications at admission). Survival was comparatively analyzed using Kaplan–Meier curves and the log-rank test. Cox proportional hazards regression analysis was performed to examine the association between polypharmacy at admission (reference: non-polypharmacy) and long-term mortality while adjusting for age, Charlson comorbidity index, and the Sequential Organ Failure Assessment (SOFA) score modeled as a continuous variable. In addition, we similarly analyzed the association between polypharmacy status at discharge among patients discharged alive and long-term mortality.

**Results:**

The study cohort comprised 533 patients (non-polypharmacy: 207 patients, polypharmacy: 326 patients). The median follow-up duration was 2.1 months (interquartile range [IQR], 0.6–11.7 months; maximum, 112.7 months). Among patients discharged alive, the median follow-up duration was 3.6 months (IQR, 1.0–19.6 months). After adjustment for age, Charlson comorbidity index, and SOFA score, patients with polypharmacy at admission were not independently associated with all-cause mortality (hazard ratio [HR]: 1.17, 95% confidence interval [CI]: 0.85–1.60). In contrast, among patients discharged alive, polypharmacy at hospital discharge showed a borderline association with increased all-cause mortality (HR, 1.67; 95% CI, 0.98–2.85).

**Conclusions:**

In critically ill older patients requiring mechanical ventilation, polypharmacy at emergency department admission was not independently associated with long-term mortality after adjustment for acute illness severity. Polypharmacy at hospital discharge showed a borderline association with increased long-term mortality, suggesting that medication burden at discharge may reflect underlying clinical vulnerability rather than a direct causal effect.

**Supplementary Information:**

The online version contains supplementary material available at 10.1186/s12873-025-01463-x.

## Background

As populations worldwide continue to age, the proportion of older adults aged ≥ 65 years is projected to steadily increase over the coming decades [1]. Due to this major shift in demographics, healthcare and welfare systems must address rapidly evolving health challenges, such as the rise in chronic diseases, declining functional independence, and excessive polypharmacy [1]. Polypharmacy, which can be defined as the concurrent use of ≥ 5 medications [2], is associated with higher risks of adverse drug reactions, drug interactions, reduced medication adherence, and duplicate prescriptions [3].

In addition to elevating the risk of various health problems at the patient level (e.g., falls, cognitive impairment, and malnutrition), polypharmacy can also increase the burden on society and healthcare systems through higher hospitalization rates and healthcare costs [4, 5]. Studies have estimated that the prevalence of polypharmacy can reach 40% among older adults aged ≥ 65 years [6].

Polypharmacy has also become an important issue in the field of emergency medicine. A population-wide study of all general intensive care units (ICUs) in Scotland reported that preadmission polypharmacy was associated with emergency readmissions [7]. Furthermore, a Japanese study demonstrated that polypharmacy increases the risk of emergency hospitalization related to adverse drug reactions [8]. Critically ill older patients who present to the emergency department often require escalation of care to the ICU, including mechanical ventilation. Accordingly, there is a need to examine the clinical outcomes associated with polypharmacy in emergency care settings, particularly among critically ill older patients. However, evidence regarding the impact of preadmission polypharmacy on long-term survival among emergency department patients requiring mechanical ventilation remains limited. As both the prevalence of polypharmacy and the need for emergency care are increasing in Japan’s aging population, it is especially important to understand this relationship in Japanese hospitals to guide medication management policies and practices.

To evaluate the potential impact of polypharmacy at emergency department admission on long-term mortality, we conducted a retrospective cohort study of critically ill older patients who required mechanical ventilation at a university hospital in Japan. In addition, we also analyzed the association between polypharmacy status at discharge and long-term mortality among patients discharged alive.

## Methods

### Study design and data sources

We conducted a single-center retrospective cohort study of critically ill patients aged ≥ 65 years who were admitted to the emergency department and received mechanical ventilation at the ICU of a large teaching hospital in Osaka, Japan. The study period was from April 1, 2015 to December 31, 2024. The data sources were electronic medical records and insurance claims data. Each patient’s medications at the time of admission and discharge were identified from the medical records.

Given the retrospective observational design of the study, the requirement for informed consent was waived in accordance with the institutional review board regulations. This study was approved by the institutional review board of Kansai Medical University Hospital (Approval number: 2024388).

### Patient selection

Patients admitted to the emergency department were identified based on specific administrative codes assigned to emergency hospital admissions under the Japanese healthcare system. Using insurance claims data, we identified those aged ≥ 65 years who received mechanical ventilation at the ICU. We excluded patients who fulfilled any of the following criteria: (i) cardiopulmonary arrest on arrival and subsequently confirmed dead, (ii) died within 24 h of admission, and (iii) missing medication data on admission. For the analysis of polypharmacy at discharge, the analysis was restricted to patients who were discharged alive.

### Patient characteristics and treatments

We identified patient demographics (e.g., age and sex), dates of admission and discharge, comorbidities, prescription medications, duration of mechanical ventilation, duration of ICU and hospital stay, and discharge status. The primary diagnoses and comorbidities were identified based on the corresponding International Classification of Diseases, 10th Revision codes recorded in the claims data. The primary diagnoses leading to emergency admission were categorized into the following groups: diseases of the digestive system; injury (K00–K93), poisoning and certain other consequences of external causes (S00–T98); diseases of the respiratory system (J00–J99); diseases of the circulatory system (I00–I99); diseases of the nervous system (G00–G99); diseases of the genitourinary system (N00–N99); and others. These classifications were applied consistently across all patients. These ICD-10–based classifications were applied consistently across all patients. The Charlson comorbidity index (CCI) was used as an indicator of chronic comorbidity burden [9, 10]. Acute illness severity was assessed using the Sequential Organ Failure Assessment (SOFA) score, which was extracted at the time of ICU admission. The SOFA score was included as a covariate in the Cox proportional hazards regression models to adjust for acute disease severity. Direct measures of frailty were not available in the present dataset.

For this study, we examined the following medications: antihypertensives, drugs for peptic ulcer, laxatives, anticoagulants, dyslipidemia drugs, hypnotics-sedatives/anxiolytics, drugs for behavioral and psychological symptoms of dementia (BPSD), anti-inflammatory analgesics, antidiabetics, anticholinergics, antimicrobials, and antidepressants.

### Exposure and outcome variables

The primary exposure was polypharmacy status at the time of admission. Patients who were concurrently using ≥ 5 regularly prescribed medications at admission were categorized into the polypharmacy group, while patients with fewer medications were categorized into the non-polypharmacy group [2, 11]. The target medications were determined based on the Japanese government’s Guidance on Appropriate Medication for Elderly Patients, and only included those taken regularly [12]. Therefore, we excluded vaccines and medications that were taken as-needed for symptom relief (e.g., topical agents, herbal remedies, inhalant drugs, and transdermal patches). The secondary exposure was polypharmacy status at the time of discharge, which was evaluated only among patients discharged alive.

The study’s outcome measure was long-term mortality. In this study, we examined the associations of polypharmacy status at admission and at discharge with this outcome.

### Statistical analysis

First, we conducted a descriptive analysis of the patients’ characteristics, treatments, and outcomes. Continuous variables were summarized as means and standard deviations or medians and interquartile ranges, as appropriate, and categorical variables were summarized as numbers and percentages. Student’s *t*-test and the Chi-squared test were used to compare the continuous and categorical variables, respectively, between the polypharmacy and non-polypharmacy groups. We plotted Kaplan–Meier survival curves to examine survival in both groups. The log-rank test was used to compare the difference in survival trends.

Next, we constructed a Cox proportional hazards regression model with long-term mortality as the dependent variable, and polypharmacy at admission (reference: no polypharmacy at admission) as the independent variable of interest. Age (years) and CCI score (1, 2, and ≥ 3), and the SOFA score modeled as a continuous variable were included as covariates. The hazard ratios (HRs) and 95% confidence intervals (CIs) were calculated for the independent variables. This analysis was repeated in patients who were discharged alive with polypharmacy at discharge (reference: no polypharmacy at discharge) as the independent variable of interest.

A two-sided p value < 0.05 was considered statistically significant. All analyses were performed using SPSS Version 30.0 (IBM Japan, Ltd., Tokyo, Japan).

## Results

**Fig. 1:**
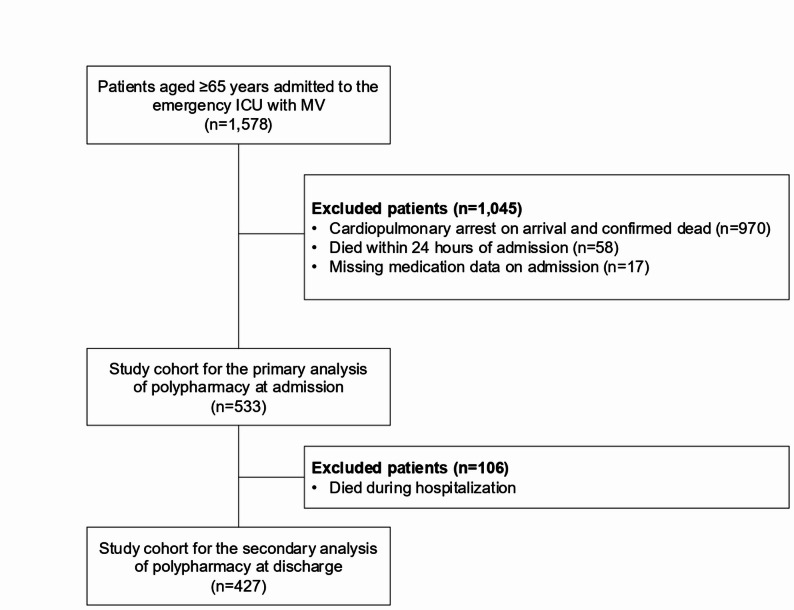
Flowchart of patient selection. *ICU* intensive care unit, *MV* mechanical ventilation

The flowchart of patient selection is presented in Fig. 1. During the study period, there were 1,578 patients aged ≥ 65 years who were admitted to the emergency department and received mechanical ventilation at the ICU. After employing the exclusion criteria, the study cohort for the primary analysis of polypharmacy at admission comprised 533 patients. The median follow-up duration was 2.1 months (interquartile range [IQR], 0.6–11.7 months; maximum, 112.7 months). Table 1 summarizes these patients’ baseline characteristics, treatments, and outcomes. Baseline SOFA scores at ICU admission were comparable between the non-polypharmacy and polypharmacy groups (Table 1).

**Table 1:** Patient characteristics, treatments, and outcomes according to polypharmacy status at admission (*n* = 533)

	Non-polypharmacy group (*n* = 207)	Polypharmacy group (*n* = 326)	*P* value
Patient characteristics			
Age (years)	77.1 ± 7.1	78.3 ± 7.0	0.07
Male	132 (63.8)	201 (61.7)	0.62
Prescribed medications at admission			
Antihypertensives	61 (29.5)	230 (70.6)	< 0.001
Drugs for peptic ulcer	34 (16.4)	212 (65.0)	< 0.001
Laxatives	18 (8.7)	141 (43.3)	< 0.001
Anticoagulants	22 (10.6)	139 (42.6)	< 0.001
Dyslipidemia drugs	21 (10.1)	125 (38.3)	< 0.001
Hypnotics-sedatives/anxiolytics	16 (7.7)	109 (33.4)	< 0.001
BPSD drugs	22 (10.6)	93 (28.5)	< 0.001
Anti-inflammatory analgesics	15 (7.2)	81 (24.8)	< 0.001
Antidiabetics	21 (10.1)	79 (24.2)	< 0.001
Anticholinergics	13 (6.3)	51 (15.6)	0.001
Antimicrobials	8 (3.9)	27 (8.3)	0.04
Antidepressants	1 (0.5)	9 (2.8)	0.05
Charlson comorbidity index			
0	133 (64.3)	174 (53.4)	0.07
1	44 (21.3)	81 (24.8)	
2	20 (9.7)	50 (15.3)	
≥3	10 (4.8)	21 (6.4)	
Processes			
Mechanical ventilation duration (days)	10.0 ± 12.5	13.0 ± 22.2	0.05
Outcomes			
ICU stay (days)	8.1 ± 8.8	8.9 ± 11.4	0.35
Overall hospital stay (days)	30.4 ± 25.1	30.3 ± 33.2	0.98
In-hospital mortality	41 (19.8)	65 (19.9)	0.97

Values are presented as mean ± standard deviation for continuous variables and number (percentage) for categorical variables

*BPSD* behavioral and psychological symptoms of dementia, *ICU* intensive care unit

There were 207 patients in the non-polypharmacy group and 326 patients in the polypharmacy group. We found no significant differences between the non-polypharmacy group and the polypharmacy group in age (77.1 ± 7.1 years vs. 78.3 ± 7.0 years, *p* = 0.07), sex distribution (men: 63.8% vs. 61.7%, *p* = 0.62), or CCI scores (*p* = 0.07) (Student’s *t*-test or the Chi-squared test, as appropriate). In the non-polypharmacy group, the most commonly prescribed medications at admission were antihypertensives (29.5%), followed by drugs for peptic ulcer (16.4%), anticoagulant drugs (10.6%), and BPSD drugs (10.6%). In the polypharmacy group, the most commonly prescribed medications at admission were antihypertensives (70.6%), drugs for peptic ulcer (65.0%), and laxatives (43.3%). The polypharmacy group had a significantly higher use of most medications except antidepressants. There were no significant differences between the non-polypharmacy group and polypharmacy group in ICU stay (8.1 ± 8.8 days vs. 8.9 ± 11.4 days, *p* = 0.35), overall hospital stay (30.4 ± 25.1 days vs. 30.3 ± 33.2 days, *p* = 0.98), or in-hospital mortality (19.8% vs. 19.9%, *p* = 0.97) (Student’s *t*-test or the Chi-squared test, as appropriate).

Table 2 shows the distribution of primary diagnoses according to polypharmacy status at admission. In both the non-polypharmacy and polypharmacy groups, diseases of the digestive system (41.1% and 40.8%); injury, poisoning and certain other consequences of external causes (27.5% and 18.1%); and diseases of the respiratory system (15.9% and 19.0%) were prevalent. There were no significant inter-group differences in these diagnoses (*p* = 0.14). Table 3 presents the distribution of prescribed medications at discharge among patients who were discharged alive (*n* = 427). The polypharmacy group had a significantly higher use of antihypertensives, drugs for peptic ulcer, anticoagulants, dyslipidemia drugs, BPSD drugs, antidiabetics, and anticholinergics.

**Table 2:** Primary diagnoses leading to emergency admission according to polypharmacy status at admission (*n* = 533)

	Non-polypharmacy group (*n* = 207)	Polypharmacy group (*n* = 326)	*P* value
Diseases of the digestive system	85 (41.1)	133 (40.8)	0.14
Injury, poisoning and certain other consequences of external causes	57 (27.5)	59 (18.1)	
Diseases of the respiratory system	33 (15.9)	62 (19.0)	
Diseases of the circulatory system	6 (2.9)	20 (6.1)	
Diseases of the nervous system	6 (2.9)	14 (4.3)	
Diseases of the genitourinary system	6 (2.9)	11 (3.4)	
Others	14 (6.8)	27 (8.3)	

Values are presented as number (percentage)

**Table 3:** Prescribed medications at discharge among patients who were discharged alive according to polypharmacy status at admission (*n* = 427)

	Non-polypharmacy group (*n* = 166)	Polypharmacy group (*n* = 261)	*P* value
Antihypertensives	46 (27.7)	111 (42.5)	< 0.01
Drugs for peptic ulcer	126 (75.9)	222 (85.1)	0.02
Laxatives	38 (22.9)	80 (30.7)	0.08
Anticoagulants	43 (25.9)	109 (41.8)	< 0.001
Dyslipidemia drugs	1 (0.6)	12 (4.6)	0.02
Hypnotics-sedatives/anxiolytics	33 (19.9)	60 (23.0)	0.45
BPSD drugs	38 (22.9)	92 (35.2)	< 0.01
Anti-inflammatory analgesics	19 (11.4)	27 (10.3)	0.72
Antidiabetics	14 (8.4)	43 (16.5)	0.02
Anticholinergics	4 (2.4)	19 (7.3)	0.03
Antimicrobials	13 (7.8)	20 (7.7)	0.95
Antidepressants	0 (0)	3 (1.1)	0.23

Values are presented as number (percentage). Polypharmacy status at discharge was unavailable for three patients

*BPSD* behavioral and psychological symptoms of dementia

**Fig. 2:**
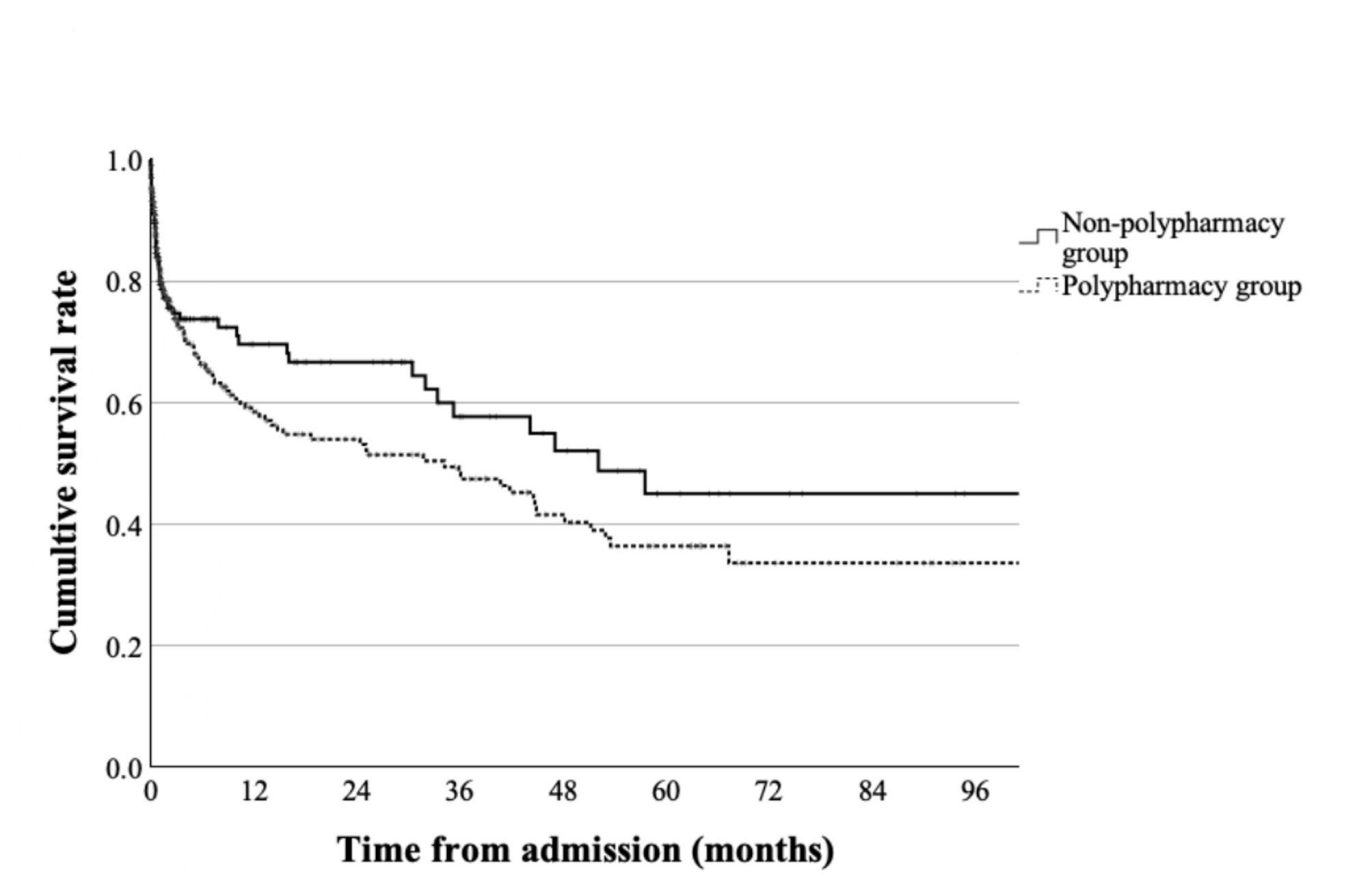
Kaplan–Meier survival curves according to polypharmacy status at admission. Before adjusting for the covariates, no significant differences were found between the non-polypharmacy group (solid line) and polypharmacy group (dotted line)

The Kaplan–Meier curves of overall survival according to polypharmacy status at admission are shown in Fig. 2. There were no significant inter-group differences in the unadjusted survival rate (log-rank test: *p* = 0.19).

**Fig. 3:**
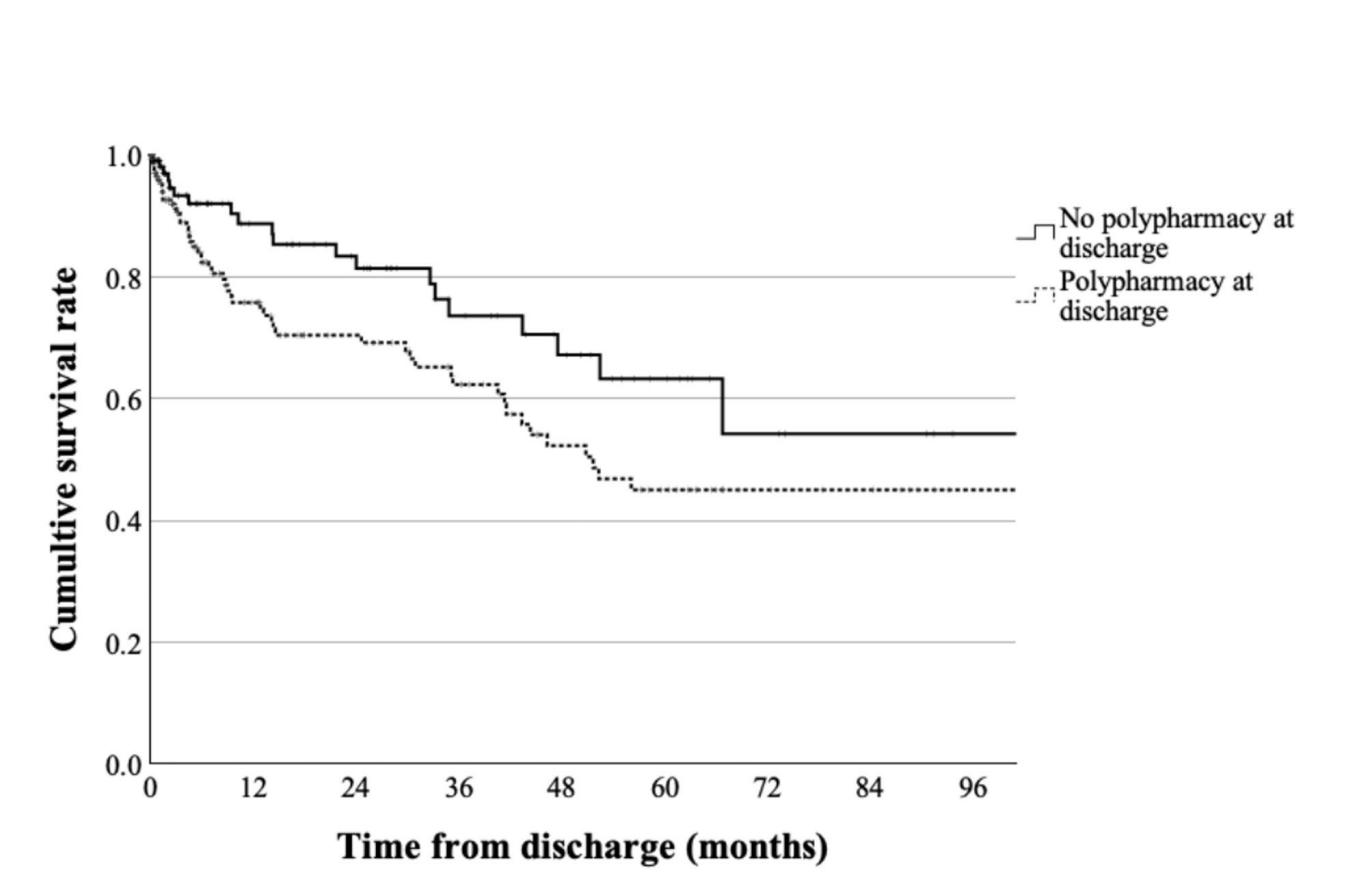
Kaplan–Meier survival curves according to polypharmacy status at discharge. Before adjusting for the covariates, no significant differences were found between patients with no polypharmacy at discharge (solid line) and patients with polypharmacy at discharge (dotted line)

The Kaplan–Meier curves of overall survival according to polypharmacy status at discharge are shown in Fig. 3. The non-polypharmacy group had a significantly higher unadjusted survival rate than the polypharmacy group (log-rank test: *p* = 0.03).

Table 4 presents the results of the Cox proportional hazards analysis of all-cause mortality according to polypharmacy status at admission. After adjustment for age, CCI, and SOFA score modeled as a continuous variable, polypharmacy at admission was not independently associated with all-cause mortality (HR: 1.17, 95% CI: 0.85–1.60). Among patients discharged alive, polypharmacy at hospital discharge showed a borderline association with increased all-cause mortality after adjustment for age, CCI, and SOFA score (HR: 1.67, 95% CI: 0.98–2.85; Supplementary Table 2).

**Table 4:** Results of the Cox proportional hazards analysis of all-cause mortality during follow-up according to polypharmacy status at admission (*n* = 533)

Variables	Hazard ratio	95% CI	*P* value
Polypharmacy group (Reference: Non-polypharmacy group)	1.17	0.85–1.60	0.34
Age	1.04	1.02–1.06	< 0.01
SOFA score	1.11	1.07–1.15	< 0.001
Charlson comorbidity index (Reference: 0)			
1	0.89	0.61–1.29	0.34
2	0.93	0.59–1.47	
≥3	1.51	0.89–2.55	

*CI* confidence interval, *SOFA* Sequential Organ Failure Assessment

The distribution of prescribed medications at discharge according to polypharmacy status at discharge is presented in Supplementary Table 1. Among patients discharged alive, the proportion of patients with polypharmacy at discharge (58.3%) was lower than that at admission (61.2%). Regardless of polypharmacy status at discharge, the most commonly prescribed medications at discharge were drugs for peptic ulcer, antihypertensives, and anticoagulants. The results of the Cox proportional hazards analysis of all-cause mortality according to polypharmacy status at discharge are presented in Supplementary Table 2.

## Discussion

In this retrospective cohort study, we examined the associations between polypharmacy, defined as the concurrent use of ≥ 5 regularly prescribed medications, and long-term mortality in critically ill older emergency patients requiring mechanical ventilation. After adjustment for age, comorbidity burden, and acute illness severity assessed using the SOFA score, polypharmacy at admission was not independently associated with long-term mortality. The lack of an independent association between polypharmacy at admission and long-term mortality is biologically plausible, as outcomes in critically ill emergency patients are strongly influenced by the severity of acute illness at presentation, which was accounted for in our analysis using the SOFA score. In contrast, among patients discharged alive, polypharmacy at hospital discharge showed a borderline association with increased all-cause mortality after adjustment for age, comorbidity burden, and acute illness severity. The observed borderline association between polypharmacy at discharge and long-term mortality should be interpreted with caution. Rather than indicating a direct causal effect, polypharmacy at discharge may reflect underlying clinical vulnerability, residual disease burden, or complexity of care among patients who survive critical illness. Previous studies have highlighted the importance of frailty-related factors in emergency department populations. For example, Demir et al. demonstrated that shorter inter-visit duration among older adults who frequently use the emergency department was associated with increased mortality, suggesting that recurrent healthcare utilization may reflect underlying clinical vulnerability [13]. Similarly, Senguldur and Selki reported high rates of critical care utilization and adverse outcomes among very old patients presenting to the emergency department, underscoring the substantial burden of frailty and complexity in this population [14]. These findings support the interpretation that markers of vulnerability, rather than single disease-specific factors, may play an important role in long-term outcomes after emergency care. Medication information at hospital discharge was clearly documented in the medical records, supporting the reliability of discharge polypharmacy assessment. Although evidence directly linking discharge polypharmacy to long-term mortality remains limited, our findings suggest that polypharmacy at discharge may serve as a clinically relevant marker of post-critical illness vulnerability and warrant further investigation.

Polypharmacy is a known risk factor for numerous adverse outcomes, such as frailty, functional decline, and death [2]. This issue is particularly important for countries with rapidly aging populations, such as Japan. However, studies have noted that emergency care settings frequently lack sufficient information on patients’ medication histories [15–17]. Under Japan’s healthcare system, prescriptions are recorded in electronic records as well as physical medication notebooks kept by the patients themselves. As this system is also used for emergency patients, the recorded data can facilitate research on medication histories and polypharmacy at this point of care.

Our study cohort was limited to emergency patients who used mechanical ventilation at admission. This decision was made with considerations to the characteristics of Japan’s emergency care system: the universal health insurance system and unrestricted access to emergency care have led to a high volume of visits to emergency departments by patients with relatively minor conditions, creating a social problem known as “convenience-store consultations” [18]. As university hospitals are also affected by this phenomenon, it was necessary for us to distinguish between patients with minor conditions and critically ill patients who truly required emergency care. To ensure that the study cohort comprised emergency patients with a clinically confirmed severe condition, we focused on those who required mechanical ventilation. In the fields of emergency and critical care research, patients requiring mechanical ventilation have been widely used as study populations [19–22]. By focusing on this patient group, we sought to minimize the impact of confounding due to the inclusion of patients with mild conditions and to strengthen the internal validity of the association between polypharmacy and long-term mortality.

Polypharmacy has been identified as an important social problem, and is reported to be associated with higher risks of falls, cognitive impairment, hospitalization, and mortality [4]. However, the definitions of polypharmacy vary among previous studies in terms of the numbers and types of target medications, and there is currently no consensus on the specific thresholds and criteria. In this study, we defined polypharmacy as a minimum of 5 regularly prescribed medications in accordance with studies by Gnjidic et al. and Masnoon et al., which have reported that this definition is valid and widely used [2, 11]. Nevertheless, it is unclear whether changing this definition would alter our observed associations between polypharmacy and long-term mortality. Our analysis showed that polypharmacy, as defined using a widely used criterion, was not significantly associated with long-term mortality. Similarly, a multicenter study in Spain found that polypharmacy, while common among older emergency patients, was not associated with 30-day mortality [23]. As our study used a longer follow-up period, these findings collectively suggest that polypharmacy at admission may not impact mortality in the short- to long-term. In contrast, van Dam et al. reported that polypharmacy increased the risk of 3-month mortality in emergency patients aged ≥ 70 years in the Netherlands [24]. This discrepancy may be due to differences in study design, clinical settings, and healthcare systems. Further research is needed to explore the association between polypharmacy and mortality under various conditions to promote a more comprehensive understanding of this relationship.

Studies have also highlighted the importance of reviewing prescriptions at discharge, and polypharmacy at discharge has become an important topic for research [3, 25, 26]. An Italian study found no association between polypharmacy (defined as the use of ≥ 8 drugs) at discharge and 1-year mortality among patients discharged from geriatric and internal medicine acute care wards [27]. This differed from our study, which found that polypharmacy (defined as the use of ≥ 5 drugs) at discharge was associated with increased long-term mortality among emergency patients. In addition to the differences in polypharmacy definition and target patients, our study’s follow-up was based on medical records without additional interviews to confirm the use of the recorded medications. Nevertheless, our observed association between polypharmacy at discharge and long-term mortality in patients who survived after receiving critical care may provide important insight to support the optimization of medication management in clinical practice.

This study has several limitations. First, this was a single-center cohort study of patients who were transported to the emergency department of a university hospital in Japan. Therefore, our findings may not be generalizable to other hospitals and populations, and large-scale multicenter studies involving a variety of hospital types are needed to verify our results. Second, our study cohort may be vulnerable to selection bias due to its focus on patients who received mechanical ventilation. While this was a deliberate decision to ensure that the patients had severe disease requiring critical care, the possibility of selection bias cannot be ruled out. Third, overall patient condition at admission was analyzed using CCI scores. The CCI was designed as a scoring system for predicting mortality based on a patient’s concurrent comorbidities. As previous studies have shown associations between CCI scores and mortality in emergency patients [28–30], we adopted this score as a measure of patient condition in our analysis of mortality in the short- to long-term. However, our study lacked certain clinical indicators of acute illness severity, such as the Acute Physiology and Chronic Health Evaluation II score, which are widely used in critical care research. Although acute illness severity was adjusted for using the SOFA score, other dimensions of frailty, such as functional status or prior healthcare utilization, were not directly available in this dataset. In addition, although medications were categorized descriptively, we did not perform analyses stratified by specific medication classes. Therefore, the potential differential effects of high-risk drug categories, such as benzodiazepines or anticholinergic agents, on long-term mortality could not be evaluated in the present study. Fourth, we could not ascertain the causes of death during the follow-up period. Unlike deaths that occur during hospitalization, medical records are not required to include detailed information on the cause of post-discharge deaths. Thus, we were unable to explore the associations between polypharmacy and cause-specific mortality. Fifth, this study used a retrospective design. Therefore, we could not determine causality in the studied relationships. While retrospective studies can be conducted with greater speed and ease, they do not allow for the comprehensive adjustment of confounders provided by prospective studies. Further studies using prospective designs are needed to determine the causal relationships between polypharmacy and long-term outcomes.

## Conclusions

In critically ill older patients requiring mechanical ventilation, polypharmacy at emergency department admission was not independently associated with long-term mortality after adjustment for acute illness severity. In contrast, polypharmacy at hospital discharge showed a borderline association with increased long-term mortality among patients discharged alive. These findings suggest that medication burden at discharge may serve as a marker of underlying clinical vulnerability rather than a direct causal factor. Careful medication review at the time of discharge may therefore be important for identifying high-risk patients after critical illness. Further studies are warranted to clarify these associations across different definitions of polypharmacy, patient populations, and healthcare settings.

## Supplementary Information

Below is the link to the electronic supplementary material.

Supplementary Material 1

Supplementary Material 2

## Data Availability

The dataset used in this study is available from the corresponding author on reasonable request.

## Abbreviations

BPSD: Behavioral and psychological symptoms of dementia
CCI: Charlson comorbidity index
CI: Confidence interval
HR: Hazard ratio
ICU: Intensive care unit
