# Supplementary material for: Association between polypharmacy at the emergency department and long-term mortality in critically ill older patients receiving mechanical ventilation: a single-center retrospective cohort study
Source: BMC Emerg Med. 2026 Jan 10;26:46. doi: 10.1186/s12873-025-01463-x (PMC12882600; doi:10.1186/s12873-025-01463-x)
Supplement: Supplementary file 1 — Supplementary Material 1 [file 12873_2025_1463_MOESM1_ESM.docx]

**Supplementary Table 1** Prescribed medications at discharge among patients who were discharged alive according to polypharmacy status at discharge (n=427)

|  | No polypharmacy at discharge  (n=178) | Polypharmacy at discharge  (n=249) | *P* value |
| --- | --- | --- | --- |
| Hypnotics-sedatives/anxiolytics | 13 (7.3) | 80 (32.1) | <0.001 |
| Antidepressants | 0 (0) | 3 (1.2) | 0.20 |
| BPSD drugs | 32 (18.0) | 98 (39.4) | <0.001 |
| Antihypertensives | 36 (20.2) | 121 (48.6) | <0.001 |
| Antidiabetics | 12 (6.7) | 45 (18.1) | <0.001 |
| Dyslipidemia drugs | 2 (1.1) | 11 (4.4) | 0.05 |
| Anticoagulants | 31 (17.4) | 121 (48.6) | <0.001 |
| Drugs for peptic ulcer | 116 (65.2) | 232 (93.2) | <0.001 |
| Anti-inflammatory analgesics | 9 (5.1) | 37 (14.9) | 0.001 |
| Antimicrobials | 9 (5.1) | 24 (9.6) | 0.08 |
| Laxatives | 33 (18.5) | 85 (34.1) | 0.08 |
| Anticholinergics | 4 (2.2) | 19 (7.6) | 0.02 |

Values are presented as number (percentage). Polypharmacy status at discharge was unavailable for three patients.

*BPSD* behavioral and psychological symptoms of dementia.
