# Supplementary material for: Association between polypharmacy at the emergency department and long-term mortality in critically ill older patients receiving mechanical ventilation: a single-center retrospective cohort study
Source: BMC Emerg Med. 2026 Jan 10;26:46. doi: 10.1186/s12873-025-01463-x (PMC12882600; doi:10.1186/s12873-025-01463-x)
Supplement: Supplementary file 2 — Supplementary Material 2 [file 12873_2025_1463_MOESM2_ESM.docx]

**Supplementary Table 2** Results of the Cox proportional hazards analysis of all-cause mortality during follow-up according to polypharmacy status at discharge (n=427)

| Variables | Hazard ratio | 95% CI | *P* value |
| --- | --- | --- | --- |
| Polypharmacy at discharge  (Reference: No polypharmacy at discharge) | 1.67 | 0.98-2.85 | 0.06 |
| Age | 1.07 | 1.03-1.11 | <0.001 |
| SOFA score | 1.05 | 0.99-1.12 | 0.14 |
| Charlson comorbidity index (Reference: 0) |  |  |  |
| 1 | 1.22 | 0.67-2.22 | <0.001 |
| 2 | 2.72 | 1.46-5.07 |  |
| ≥3 | 3.57 | 1.70-7.48 |  |

*CI* confidence interval, *SOFA* Sequential Organ Failure Assessment.
